# DOA Estimation of Unknown Emitter Signal Based on Time Reversal and Coprime Array

**DOI:** 10.3390/s19061398

**Published:** 2019-03-21

**Authors:** Bing Li, Shiqi Liu, Deshuang Zhao, Bin-Jie Hu

**Affiliations:** 1 School of Electrical Engineering, Southwest Jiaotong University, Chengdu 610031, China; 2 School of Physics, University of Electronic Science and Technology of China, Chengdu 610054, China; bllijess@outlook.com or dszhao@uestc.edu.cn; 3 Southwest China Research Institute of Electronic Equipment, Chengdu 610036, China; liu.shiqi@outlook.com; 4 School of Electronic and Information Engineering, South China University of Technology, Guangzhou 510641, China; eebjiehu@scut.edu.cn

**Keywords:** coprime array, direction-of-arrival, low signal-to-noise ratio, multipath, noise suppression, time reversal

## Abstract

In this paper, a novel direction-of-arrival (DOA) estimation for unknown (anonymous) emitter signal (ES) based on time reversal (TR) and coprime array (CA) is proposed. The resolution and accuracy of DOA estimation are enhanced from two aspects: one is from the view of array arrangement: the new distribution of CA is designed to reduce the holes, increase the degree of freedom (DOF) and apertures by rotating and translating only one subarray, which simplifies the operation. The other one is from the view of the algorithm: a neoteric DOA estimation algorithm with noise suppression based on TR, Capon and adaptive neuro-fuzzy inference system (ANFIS) is proposed for solving the wide sidelobe, multipath effect, low resolution and accuracy produced by conventional algorithms, in particular, those cannot work effectively under the existed hole condition. Furthermore, the resubmitting distorted noise and channel noise are suppressed effectively, which is not taken into considered in the conventional Capon algorithm. Simulation results including the resolution, accuracy, root mean square error (RMSE), Cramér-Rao lower bound (CRLB) and the compared analyses on uniform linear array (ULA), nested array (NA) and minimum redundancy array(MRA) demonstrate the performance advantages of the proposed DOA estimation algorithm even at very low signal-to-noise ratio (SNR) condition.

## 1. Introduction

Presently, with the continuous appearance of new wireless communication and position systems, locating the emitter signal (ES) plays a more and more important role in public security, fraud detection, and intelligent transportation systems [1,2,3]. Moreover, because of the ever-increasing number of different emitters and waveforms as well as increasing data processing demands, the location technique for ES becomes more difficult than before [4,5] and very essential. Based on this, direction of arrival (DOA) estimation becomes a good candidate for precise attainment of ES direction and persuades considerable contemplation of researchers for years [6,7]. Recent years, there are many DOA estimation methods springing up, such as estimation of signal parameter via rotational invariance technique (ESPRIT) [8], multiple signal classification (MUSIC) [9] and decomposition of reverse time operator (DORT) [10]. However, these subspace methods present a high complexity due to the fact that they strongly rely on eigenvalues or singular value decomposition for differentiating the signal or noise subspace. Besides, these subspace methods have to calculate the sample covariance matrix every snapshot, which leads to a high amount of internal processing. While, compared with these algorithms, Capon [11] algorithm has more superiority in DOA estimation [12,13,14]. Besides, Most DOA estimation algorithms attempt to eliminate the effect of multipath using deconvolution with the approximated channel impulse response or channel equalization, which treats multipath as clutter or noise. These approaches result in the loss of some useful information on ES, and the resolution and accuracy are limited. Unlike these methods, one of effective approaches to address this problem is to take advantage of time reversal (TR) technology which performs a good robustness in rich multipath environment and treats multipath as useful echo [15,16,17]. Thus, the improved Capon DOA estimation algorithm based on TR is researched in this paper.

Furthermore, the performance of antenna array is also able to be optimized pertaining to enhancing the resolution and accuracy of detecting sources. An effective way is to increase the number of degrees of freedom (DOF) by designing a large aperture array [18]. Higher DOF means more sources can be recognized. Thus, one of the design crux is to acquire as high DOF as possible. In addition, resolution will be deteriorated under low signal-to-noise ratio (SNR) condition [19,20]. Therefore, noise suppression is another key aspect needed to be considered as well.

### 1.1. DOF Design and Method of Increasing Effective Aperture of Array for DOA Estimation

**Figure 1:**
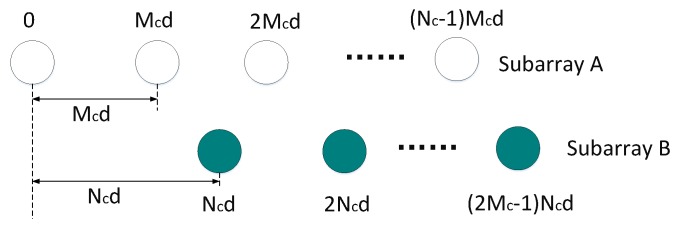
Elements’ positions of the CA.

Various non-uniform linear antenna (ULA) arrays are designed for increasing DOF and effective apertures of array. These methodologies have been reported in the literature, such as coprime array (CA) [21,22]. As shown in Figure 1, a conventional CA consists of a coprime pair of uniform linear subarrays (ULSAs) with $2M_{c}$ and $N_{c}$ elements, where $M_{c}$ and $N_{c}$ are coprime. The unit spacing between two consecutive elements is $N_{c}d$ in the subarray B, while the unit spacing between two consecutive elements is $M_{c}d$ in the other ULSA, therein *d* is typically set to λ/2, and λ is the wavelength of impinging narrowband ES. These two ULSAs are share the same first element namely the element positioned “0”. Thus, we can educe that the total number of the elements of CA is $2M_{c}+N_{c}-1$ and the elements’ position set is $S=\left\{M_{c}n_{c}d,0\leq n_{c}\leq N_{c}-1\right\}\cup \left\{N_{c}m_{c}d,1\leq m_{c}\leq 2M_{c}-1\right\}$. According to the property of coprime positive integers reported in [23], the $k_{d}$ can be chosen any integer between $-M_{c}N_{c}$ and $M_{c}N_{c}$. In other words, based on $2M_{c}+N_{c}-1$ elements, the corresponding array has $\left(2M_{c}N_{c}+1\right)$ DOF which denotes the number of consecutive elements, and $2\left(2M_{c}-1\right)N_{c}+1=4M_{c}N_{c}-2N_{c}+1$ apertures without considering the holes. As a result, an array with considerably larger aperture is achieved using coprime arrangement, compared with that generated by actual number of elements and that constructed by ordinary ULA with the equal number of elements.

However, the created large aperture virtual array is not a filled ULA (it exists some holes), which will leads other problems in DOA estimation, for example, MUSIC and DORT do not outfit in the CA encompassing holes condition [21], because they need to acquire eigenvalues and eigenvectors from covariance matrix generated by the recorded data, and the corresponding virtual array from the covariance matrix must be full rank [18,23]. Moreover, the case that just consider the consecutive virtual array elements and ignore the discontinuous elements of virtual array will lead the loss of virtual apertures. Therefore, new investigations on DOA estimation algorithm based on CA are significant and meaningful. Recovering as many holes as possible [24,25] is one method. For example, Mahmum et al. [21] translocated one subarray and rotated axis with a compression of the other subarray for producing plenty of consecutive lags, which can be used to obtain high DOF; array interpolation algorithm [24,25,26] and sparse recovery algorithm [27] are used to exploit all the information in CA and reduce the negative influence of holes. However, the sparse recovery method can increase the recoverable sparsity level only under the assumption that the received data are zero-mean, statistically uncorrelated random variables [28,29,30,31]. Thus, when it comes to unknown ES with unknown mean and correlation between each other, this algorithm is not suitable. Additionally, the aforementioned techniques need extra tuned parameters which are difficult to maintain.

Although the number of holes can be reduced, the array is still not filled. Thus, the key to solving this problem is to develop a new DOA estimation algorithm based on CA, namely, this new algorithm is suitable for CA (especially has holes) and so forth. Besides, increasing the number of DOF of CA is a good choice for enhancing the resolution and accuracy of DOA estimation. Thus, the other problem solved in this paper is to achieve an optimal method of high DOF with easy operation for CA.

### 1.2. High Resolution and Accuracy Algorithms for DOA Estimation

To enhance the resolution and accuracy of DOA estiomation, in addition to optimizing the arrangement of the array, improving the performance of DOA estimation algorithm is another effective way. For obtaining useful transmission and reflection parts of ES, and forbidding the negative effect of multipath diversity on DOA estimation, this paper considers TR as a good candidate, because TR is able to take advantage of multipath which is recognized as clutter or noise and ignored/mitigated in other most DOA estimation algorithms, such as MUSIC, DORT, back projection [32,33]. In the basic procedure of TR [15,17,34,35,36], these recorded signals from observation space are time-reversed, energy normalized and retransmitted (can be achieved numerically or actually) through the same medium from the same receivers (act as transmitters in this stage). Due to the spatial reciprocity principle, space and time focusing will occur at the location of original emitter [37]. Thus, the corresponding focusing amplitude or time can be used to locate original emitter. Furthermore, based on the TR algorithm, multistatic data matrix which is prerequisite in MUSIC, DORT is not necessary. Therefore, it can be concluded that TR is able to be operated in the CA (has holes) condition. However, compared with the general ULA, CA with the equal number of array elements has larger apertures. Although it is able to increase the useful received signal, it also increases the noise information which may result in a reduction of SNR on the contrary [38]. Thus, suppressing noise and enhancing SNR are another key problems to be solved in order to improve the resolution and accuracy of DOA estimation. Faced with the random and irregular noise, an effective way to suppress noise is to use adaptive noise suppression methods. Here, we believe that adaptive neuro-fuzzy inference system (ANFIS) is a good choice because of its outstanding performance in noise cancellation for image reported in [39,40,41]. However, they assume the information signal (original emitter) is zero mean, which is not suitable for all situations, especially for unknown ES with unknown mean. Thereupon, we take the pre-tested detection echo as the initial value of noise. These noise will be trained and cancelled through ANFIS, combined with the property that noise discussed here is background noise, namely the noise is Gaussian distribution with zero mean and uncorrelated with the useful signal.

### 1.3. Contributions of This Paper

In order to enhance the resolution and accuracy of DOA estimation, the main contributions of this paper can be summarized as the following two points: from the view of array arrangement, we design a virtual large aperture linear array based on CA; from the view of algorithm, we proposed a noise suppression DOA estimation algorithm based on TR and Capon. The detailed contributions of this paper are as follows:

(1) An optimized CA (OCA) with higher DOF is designed. By properly designing the inter-space between elements of only one subarray, which is easy to operate, a large aperture array can be obtained.
(2) For the sake of solving the problem of wide sidelobe and multipath effect, a DOA estimation algorithm based on TR and Capon is proposed (called TR-Capon-DOA algorithm here) for passive array to detect active targets. Furthermore, on the basis of TR-Capon-DOA algorithm, and in order to reduce the negative influence of noise on locating ES, a DOA estimation method with noise suppression is developed (called TR-NS-Capon-DOA here), combined with ANFIS. In the ANFIS, the distorted noise in the resubmitting stage and channel noise are considered.
(3) TR-NS-Capon-DOA, TR-Capon-DOA with the conventional counterpart–Capon algorithm are compared. The performance of these DOA estimation algorithms with ULA, CA, OCA, NA and MRA are analyzed for locating different unknown ES from different directions under the conditions of a multipath environment. Moreover, the corresponding root mean square error (RMSE), Cramér-Rao lower bound (CRLB) and computational complexity are also discussed.

### 1.4. Organizaton of This Paper

The remainder of this paper is organised as follows. Section 2 provides the system model and methodology used throughout this paper. A comparative analysis of TR-NS-Capon-DOA, TR-Capon-DOA and Capon theory is also presented in Section 2. The corresponding numerical experiments and analyses are discussed in Section 3. Finally, Section 4 concludes this paper. Moreover, Table 1 and Table 2 respectively summarize the abbreviations and the meaning of symbol and notation used in this paper, Table 3 summarizes the whole process.

## 2. System Model and Methodology

In this section, we introduce the proposed OCA first. Then, the proposed DOA estimation theory based on TR and Capon is dissected. Thereafter, in order to improve the performance of DOA estimation further, the noise suppression is considered and TR-NS-Capon-DOA algorithm combined with ANFIS is proposed. Therein, the principle of ANFIS and its function are expatiated. It is worth mentioning that the distorted noise happening in the resubmitting stage is figured out. At last, the performance of these proposed methods including suppressing noise is analyzed compared with the conventionally typical DOA estimation algorithm–Capon.

### 2.1. DOF Design and Method of Increasing Effective Aperture of Array for DOA Estimation

The layout of conventional CA is shown in Figure 1. For example, we choose $M_{c}=3$ and $N_{c}=8$ as the coprime integers, it can be found that $M_{c}$ and $N_{c}$ satisfy $M_{c}<N_{c}$. Moreover, the total number of array elements is $N=2M_{c}+N_{c}-1=13$. Generally, the conventional CA was proposed to actualize a longer consecutive virtual ULA with larger aperture from the difference copime array (DCA). The positions of corresponding DCA generated by this configuration can be expressed as

(1) $$\begin{array}{cc} S_{DCA} & =\{\pm \left(n_{c}M_{c}d-m_{c}N_{c}d\right)\}\\ & \left(0\leq n_{c}\leq N_{c}-1\right),\left(0\leq m_{c}\leq 2M_{c}-1\right) \end{array}$$

**Figure 2:**
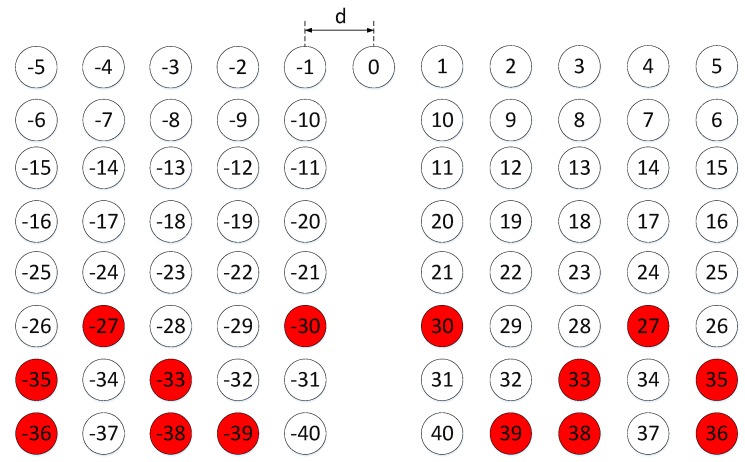
The distribution of the conventional DCA. Therein, the red circle represents the hole, and the white circle indicates the element of virtual ULA constructed by DCA.

According to [23], the corresponding number of consecutive elements is at least $2M_{c}N_{c}+1=49$, namely DOF = 49. In this case the number of effective DOF is 53 shown in Figure 2. Note that the CA and OCA are both 1-D linear arrays. In addition, there are $4M_{c}N_{c}-2N_{c}+1=81$ apertures with 14 holes appearing at labelled red positions also shown in Figure 2. Thus, the number of effective apertures is 67. During the analysis, we find some elements’ positions are calculated more than once, and the holes are never calculated. Based on this finding, in order to obtain consecutive elements as many as possible on the premise of unchanging the total number of element, a new method is proposed here to enhance the DOF for achieving the high resolution and accuracy. Firstly, rotate the axis of subarray A from positive to negative, that is, the positions of subarray A is changed into $\left[-\left(N_{c}-1\right)M_{c}d,-\left(N_{c}-2\right)M_{c}d,\cdots ,M_{c}d,0\right]$, so that the position of the maximum aperture is able to be extended to $\left[\left(N_{c}-1\right)M_{c}+\left(2M_{c}-1\right)N_{c}\right]$, which is always lager than that of original DCA-$max(\left(N_{c}-1\right)M_{c},\left(2M_{c}-1\right)N_{c})$. Then, translate the elements’ positions of subarray A by the factor-FA which can be optimized by

(2) $$arg\;\max_{FA}\;g\left(-A_{dis}+FAd,B_{dis}\right)$$

where $A_{dis}$ and $B_{dis}$ represent the position matrices of subarrays A and B respectively, and function g(a,b) is denoted to compute the maximum consecutive value of matrix (a−b), which corresponds to the number of the consecutive elements of DCA. Also, the translation factor-FA of subarray A can be obtained by calculating the optimal value of Equation (2). It is easy to summarize that the relative higher DOF can be obtained after optimizing. Besides, we do not take any operations on subarray B. Thus, the operation is simplified without changing the arrangements of both subarrays simultaneously. Furthermore, the closed-form expression for the array configuration of the proposed OCA is $S_{OCA}=\left\{\left[-M_{c}n_{c}+FA\right]d,0\leq n_{c}\leq N_{c}-1\right\}\cup \left\{N_{c}m_{c}d,0\leq m_{c}\leq 2M_{c}-1\right\}$. Thus, we can obtain the positions of new DCA as

(3) $$\begin{array}{cc} S_{DCA} & =\{\pm \left(-n_{c}M_{c}d+FAd-m_{c}N_{c}d\right)\}\\ & \left(0\leq n_{c}\leq N_{c}-1\right),\left(0\leq m_{c}\leq 2M_{c}-1\right) \end{array}$$

**Figure 3:**
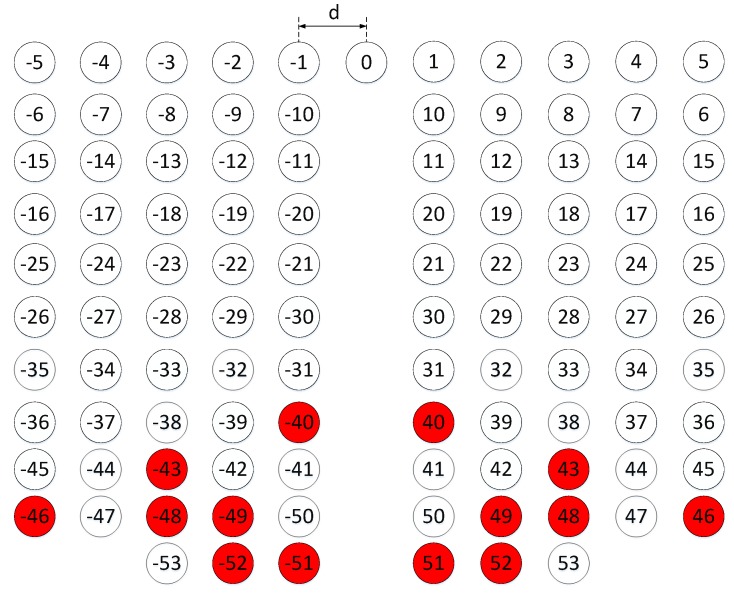
The distribution of the proposed DCA. Therein, the red circle represents the hole, and the white circle indicates the element of virtual ULA constructed by DCA.

Here, we also take $M_{c}=3$ and $N_{c}=8$ for example. According to Equation (2), the optimal FA = 8, and the new distribution of DCA is shown in Figure 3. In this construction, the number of effective DOF is 79, which is larger than that built by the conventional DCA. Moreover, there are 107 apertures also with 14 holes and the number of effective apertures is 93, which is 26 more than the original value. Furthermore, the number of holes before position “40” is 2, which is smaller than that of original DCA. In other words, compared with original DCA, although the total number of holes is the same, larger aperture is able to be achieved using the optimized OCA. In addition, from the view of obtaining the same aperture, the number of holes is reduced using the proposed optimized DCA arrangement.

For comparison, the minimum redundancy array (MRA) and nested array (NA) are researched. As we know, there are no closed-form expressions for the positions of elements in an MRA. While, the positions of elements can be figured out with exhaustion method. Here, the total number of antenna elements is N=13. In addition, literature [42] reports the minimum redundancy will lie between 1.217 and 1.332. Therefore, the maximum number of apertures (greatest multiple of the unit spacing) is 64, when N=13 and minimum redundancy is 1.218. Note that the layout of MRA is not unique, and the spacing configuration of MRA used in this paper can be adopted as {0,1,2,3,8,8,8,8,5,5,7,7,2} according to A.T. Moffet who invented this configuration of array [42]. In addition, An NA generated by the parameter pair $\left(2M_{c},N_{c}\right)$ is given by $\left\{0,1,\cdots ,2M_{c}-1\right\}\cup \left\{2M_{c},4M_{c}+1,\cdots ,2N_{c}M_{c}-1\right\}$ [9]. Therefore, the position sets consist of NA and MRA are as follows: (1). NA(5,8):[0,1,2,3,4,5,11,17,23,29,35,41,47]d, and the corresponding position set of difference array is [−47, −46, −45, −44, −43, −42, −41, −40, −39, −38, −37, −36, −36, −35, −34, −33, −32, −31, −30, −30, −30, −29, −28, −27, −26, −25, −24, −24, −24, −24, −23, −22, −21, −20, −19, −18, −18, −18, −18, −18, −17, −16, −15, −14, −13, −12, −12, −12, −12, −12, −12, −11, −10, −9, −8, −7, −6, −6, −6, −6, −6, −6, −6, −5, −4, −4, −3, −3, −3, −2, −2, −2, −2, −1, −1, −1, −1, −1, 0, 0, 0, 0, 0, 0, 0, 0, 0, 0, 0, 0, 0, 1, 1, 1, 1, 1, 2, 2, 2, 2, 3, 3, 3, 4, 4, 5, 6, 6, 6, 6, 6, 6, 6, 7, 8, 9, 10, 11, 12, 12, 12, 12, 12, 12, 13, 14, 15, 16, 17, 18, 18, 18, 18, 18, 19, 20, 21, 22, 23, 24, 24, 24, 24, 25, 26, 27, 28, 29, 30, 30, 30, 31, 32, 33, 34, 35, 36, 36, 37, 38, 39, 40, 41, 42, 43, 44, 45, 46, 47]*d*, thus, DOF = 169; (2). MRA 13 [64]: [0,1,3,6,14,22,30,38,43,48,55,62,64]d, and the corresponding position set of difference array is [−64, −63, −62, −61, −61, −59, −58, −56, −55, −54, −52, −50, −49, −48, −48, −47, −45, −43, −42, −42, −42, −41, −40, −40, −38, −37, −37, −35, −34, −34, −33, −32, −32, −30, −29, −29, −27, −26, −26, −25, −24, −24, −24, −22, −21, −21, −21, −19, −19, −18, −17, −16, −16, −16, −16, −14, −14, −13, −13, −12, −11, −10, −9, −8, −8, −8, −8, −7, −7, −6, −5, −5, −5, −3, −3, −2, −2, −1, 0, 0, 0, 0, 0, 0, 0, 0, 0, 0, 0, 0, 0, 1, 2, 2, 3, 3, 5, 5, 5, 6, 7, 7, 8, 8, 8, 8, 9, 10, 11, 12, 13, 13, 14, 14, 16, 16, 16, 16, 17, 18, 19, 19, 21, 21, 21, 22, 24, 24, 24, 25, 26, 26, 27, 29, 29, 30, 32, 32, 33, 34, 34, 35, 37, 37, 38, 40, 40, 41, 42, 42, 42, 43, 45, 47, 48, 48, 49, 50, 52, 54, 55, 56, 58, 59, 61, 61, 62, 63, 64]*d*, thus, DOF = 5 × 2 + 13 = 23. It can be seen that many antenna elements are placed so near that the mutual coupling effects raise. Hence, although the DOF in the proposed OCA case is lower than that in the NA case, the performance of DOA estimation using NA is not necessarily superior to that using OCA, which will be proved by simulation. Additionally, the DOF of MRA is lower than that of the proposed OCA, thus, the performance obtained by MRA is worse than that acquired by OCA.

### 2.2. High Resolution and Accuracy Algorithm for DOA Estimation

**Figure 4:**
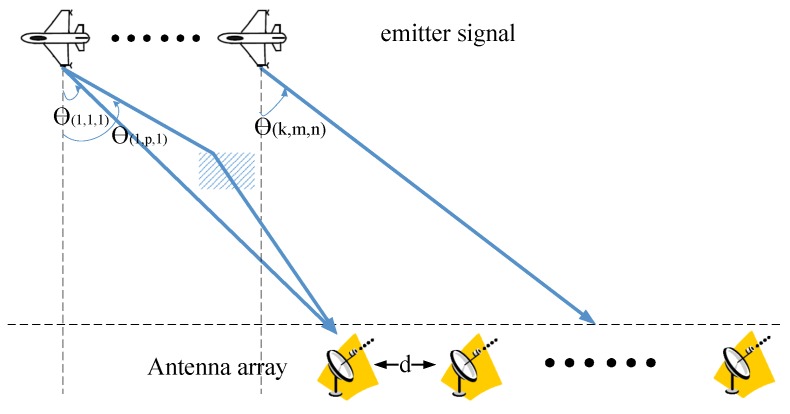
System model for DOA estimation. Background scatter is denoted by rectangle.

The system model and setup of our proposed approaches are shown in Figure 4. The system model operates in a rich multipath environment. Because of multipath, a passive antenna array receives and records the superposition of several delayed and attenuated replicas conformed by the signal from one or several ESs. Assume the signals have no relative motion, and the position and geometry of array are known. Let *K* uncorrelated narrowband ESs from directions $\theta =\left[\theta_{1},\theta_{2},\cdots ,\theta_{K}\right]=\left[\theta_{\left(1,1,1\right)},\cdots ,\theta_{\left(1,M_{\left(1,N\right)},N\right)},\theta_{\left(2,1,1\right)},\cdots ,\theta_{\left(2,M_{\left(2,N\right)},N\right)},\cdots ,\theta_{\left(K,1,1\right)},\cdots ,\theta_{\left(K,M_{\left(K,N\right)},N\right)}\right]$ impinge on the array. Notation $\theta_{\left(k,m,n\right)}$ is the DOA from ES *k* traveling via path *m* to antenna *n*, corresponding delay and attenuation are denoted by $\tau_{\left(k,m,n\right)}$ and $A_{\left(k,m,n\right)}$ respectively. $M_{\left(k,n\right)}$ represents the total number of paths between ES *k* and antenna *n*.

The 1≤k≤Kth ES $f_{k}\left(t\right)$ (with Fourier transform of $F_{k}\left(\omega \right)$) is recorded by all array elements after propagating through multipath environment with random medium. The recorded sum signal from *K* ESs at antenna *n* (1≤n≤N) is given by

(4) $$r_{n}\left(t\right)=\sum_{k=1}^{K}\sum_{m=1}^{M_{\left(k,n\right)}}A_{\left(k,m,n\right)}f_{k}\left(t-\tau_{\left(k,m,n\right)}\right)+v_{\left(k,m,n\right)}\left(t\right)$$

where t=1,2,⋯,Q, *Q* denotes the total number of snapshots; $v_{\left(k,m,n\right)}\left(t\right)$ (with Fourier transform of $V_{\left(k,m,n\right)}\left(\omega \right)$) is additive white Gaussian noise (AWGN), which is a good candidate to simulate background noise and $v_{\left(k,m,n\right)}\left(t\right)∼N\left(0,\sigma_{\left(k,m,n\right)}^{2}\right)$ [15] used as observation noise here. Note that the noise is unrelated and independent of the path and source, and the subscript (k,m,n) here is used to point out the channel where noise exists.

In the frequency domain, Equation (4) can be expressed as

(5) $$R_{n}\left(\omega \right)=\sum_{k=1}^{K}\sum_{m=1}^{M_{\left(k,n\right)}}A_{\left(k,m,n\right)}F_{k}\left(\omega \right)e^{-j\omega \tau_{\left(k,m,n\right)}}+V_{\left(k,m,n\right)}\left(\omega \right)$$

Using matrix notation, Equation (5) can be rewritten as

(6) $$R_{n}\left(\omega \right)=\left[A_{n}F\Gamma_{n}+V_{n}\right]I$$

where $A_{n}=\left[A_{\left(1,1,n\right)},A_{\left(1,2,n\right)},\cdots ,A_{\left(K,M_{\left(K,n\right)},n\right)}\right]$ is defined as a $\left(1\times \sum_{k=1}^{K}M_{\left(k,n\right)}\right)$ vector containing all attenuation factors of paths between all ESs and antenna *n* and can be obtained by log-normal shadowing model [43]. $F=diag[\overset{M_{\left(1,n\right)}}{\overbrace{F_{1}\left(\omega \right),\cdots ,F_{1}\left(\omega \right)}},\overset{M_{\left(2,n\right)}}{\overbrace{F_{2}\left(\omega \right),\cdots ,F_{2}\left(\omega \right)}},\cdots ,\overset{M_{\left(K,n\right)}}{\overbrace{F_{K}\left(\omega \right),\cdots ,F_{K}\left(\omega \right)}}]$ is defined as a $\left(\sum_{k=1}^{K}M_{\left(k,n\right)}\times \sum_{k=1}^{K}M_{\left(k,n\right)}\right)$ diagonal matrix containing all ESs, $\Gamma_{n}=diag\left[e^{j\omega \tau_{\left(1,1,n\right)}},e^{j\omega \tau_{\left(1,2,n\right)}},\cdots ,e^{j\omega \tau_{\left(K,M_{\left(K,n\right)},n\right)}}\right]$ as a $\left(\sum_{k=1}^{K}M_{\left(k,n\right)}\times \sum_{k=1}^{K}M_{\left(k,n\right)}\right)$ diagonal matrix of delays can be obtained by finite-difference time-domain (FDTD) method [44] (here $\tau_{ref}=\tau_{\left(k,M_{\left(k,1\right)},1\right)}$) combining with antenna theory [45], therein $\tau_{\left(k,M_{\left(k,n\right)},n\right)}=\frac{d_{n}sin\theta_{\left(k,M_{\left(k,n\right)},n\right)}}{c}+\tau_{ref}$, $d_{n}$ represents the distance between antenna *n* and antenna 1 (reference element) and can be obtained according to the array arrangement, $V_{n}=\left[V_{\left(1,1,n\right)},V_{\left(1,2,n\right)},\cdots ,V_{\left(K,M_{\left(K,n\right)},n\right)}\right]$ as a $\left(1\times \sum_{k=1}^{K}M_{\left(k,n\right)}\right)$ expresses vector of noise, and I is a $\left(\sum_{k=1}^{K}M_{\left(k,n\right)}\times 1\right)$ unit vector. Thus, we can get $R\left(\omega \right)={\left[R_{1}\left(\omega \right),R_{2}\left(\omega \right),\cdots ,R_{N}\left(\omega \right)\right]}^{T}$, which is defined as a (N×1) vector representing recorded signal of array.

#### 2.2.1. Conventional Capon DOA Estimation

The conventional Capon algorithm can be considered as an optimizer that attempts to maintain a fixed power while rejecting the noise and clutter maximally in the direction from the signal of interest. Therefore, the weight vector W can be obtained by solving the solution to the following minimization problem:

(7) $$\left\{\begin{array}{c} \min_{W}=W^{H}\left(\omega ,\theta \right)B(\omega ,\theta )W\left(\omega ,\theta \right)\\ s.t.W^{H}\left(\omega ,\theta \right)a\left(\omega ,\theta \right)=1 \end{array}\right.$$

where $B\left(\omega ,\theta \right)=E[R\left(\omega \right)R^{H}\left(\omega \right)]$, the steering vector $a\left(\omega ,\theta \right)=\left[e^{j\varpi_{1}sin\theta },e^{j\varpi_{2}sin\theta },\cdots ,e^{j\varpi_{N}sin\theta }\right]$ is (N×1) dimension, therein, $\varpi_{n}=\frac{\omega d_{n}}{c}$ without $\tau_{ref}$ which has no influence to the weight vector W and the optimal θ. The solution to (7) is

(8) $$W\left(\omega ,\theta \right)=\frac{B^{-1}\left(\omega ,\theta \right)a\left(\omega ,\theta \right)}{a^{H}\left(\omega ,\theta \right)B^{-1}\left(\omega ,\theta \right)a\left(\omega ,\theta \right)}$$

Thus, substituting (8) to (7), we can get the power spectrum as

(9) $$P\left(\omega ,\theta \right)=\frac{1}{a^{H}\left(\omega ,\theta \right)B^{-1}\left(\omega ,\theta \right)a\left(\omega ,\theta \right)}$$

At a specific frequency, several frequencies, a specific frequency range, or several frequency bands, the DOAs can be obtained by selecting *K* values of θ corresponding to *K* maximal values of P(ω,θ).

#### 2.2.2. TR-Capon-DOA Estimation

According to the principle of TR [37], time reverse the recorded signal $r_{n}\left(t\right)$ in time domain is equivalent to take phase conjugated operation on R(ω) in frequency domain. Therefore, the time reversed representation of $R_{n}\left(\omega \right)$ can be expressed as $R_{n}^{*}\left(\omega \right)$. Assuming this TR signal is numerically resubmitted to the same multipath environment. The rebroadcasting signal at the $k_{x}$th $\left(1\leq k_{x}\leq K\right)$ ES position from the *n*th antenna element is given by

(10) $$R_{TR_{nk_{x}}}\left(\omega \right)=\sum_{m_{x}=1}^{M_{\left(k_{x},n\right)}}g_{n}A_{\left(k_{x},m_{x},n\right)}e^{-j\omega \tau_{\left(k_{x},m_{x},n\right)}}R_{n}^{*}\left(\omega \right)+V_{\left(k_{x},m_{x},n\right)}\left(\omega \right)$$

where $g_{n}=\sqrt{\frac{∥\max_{k=1}^{K}F_{k}{\left(\omega \right)∥}^{2}}{∥R_{n}{\left(\omega \right)∥}^{2}}}$ is used as energy normalization factor. The Equation (10) can be rewritten as Equation (11).

(11) $$\begin{array}{cc} & R_{TR_{nk_{x}}}\left(\omega \right)\\ = & \sum_{m_{x}=1}^{M_{\left(k_{x},n\right)}}g_{n}A_{\left(k_{x},m_{x},n\right)}e^{-j\omega \tau_{\left(k_{x},m_{x},n\right)}}\left[\sum_{k=1}^{K}\sum_{m=1}^{M_{\left(k,n\right)}}A_{\left(k,m,n\right)}e^{j\omega \tau_{\left(k,m,n\right)}}F_{k}^{*}\left(\omega \right)+V_{\left(k,m,n\right)}^{*}\left(\omega \right)\right]+V_{\left(k_{x},m_{x},n\right)}\left(\omega \right)\\ = & \underset{useful-signal}{\underbrace{\left[g_{n}\sum_{m_{x}=1}^{M_{\left(k_{x},n\right)}}|A_{\left(k_{x},m_{x},n\right)}{|}^{2}F_{k_{x}}^{*}\left(\omega \right)\right]}}+\underset{noise1}{\underbrace{\left[g_{n}\sum_{m_{x}=1}^{M_{\left(k_{x},n\right)}}A_{\left(k_{x},m_{x},n\right)}e^{-j\omega \tau_{\left(k_{x},m_{x},n\right)}}V_{\left(k_{x},m_{x},n\right)\left(\omega \right)}^{*}\right]}}\\ & +g_{n}\underset{clutter1}{\underbrace{\sum_{m_{x}=1}^{M_{\left(k_{x},n\right)}}\sum_{\begin{array}{c} k=1\\ k\neq k_{x} \end{array}}^{K}\sum_{m=1}^{M_{\left(k,n\right)}}A_{\left(k_{x},m_{x},n\right)}A_{\left(k,m,n\right)}e^{-j\omega (\tau_{\left(k_{x},m_{x},n\right)}-\tau_{\left(k,m,n\right)})}F_{k}^{*}\left(\omega \right)}}\\ & +g_{n}\underset{clutter2}{\underbrace{\sum_{m_{x}=1}^{M_{\left(k_{x},n\right)}}\sum_{\begin{array}{c} k=1\\ k\neq k_{x} \end{array}}^{K}\sum_{m=1}^{M_{\left(k,n\right)}}A_{\left(k_{x},m_{x},n\right)}e^{-j\omega \tau_{\left(k_{x},m_{x},n\right)}}V_{\left(k,m,n\right)}^{*}\left(\omega \right)}}+\underset{noise2}{\underbrace{\sum_{m_{x}=1}^{M_{\left(k_{x},n\right)}}V_{\left(k_{x},m_{x},n\right)}\left(\omega \right)}} \end{array}$$

It can be found that the rebroadcasting signal focuses at the original ES position, namely the signal from the $k_{x}$th ES in the receiving stage focuses on the $k_{x}$th ES position in the resubmitting stage, which constructs the useful signal. Although there are clutter conformed by the resubmitting signal from other paths, and noise built by transmission environment, the DOA of the $k_{x}$th ES can be obtained by enough elements. Besides, if the $k_{x}$th emitter source does not radiate electronmagnetic wave in the receiving stage, there is no focusing phenomenon appearing at the $k_{x}$th ES position in the resubmitting process. In other words, the focusing will not happen at other non-ES’s positions. According to Equation (11), we can conclude that with the complementary time delay, TR is able to achieve channel matching, which is equivalent to beamforming used in signal processing for array. Besides, the focused performance will be better by befittingly increasing the number of multipath. Note that because the attenuation must be considered, the number of multipath is not the more the better. In summary, in the light of the advantages of TR as described above, we propose TR-DOA estimation method to enhance the resolution and accuracy of DOA estimation.

Using matrix notation, and based on Equations (10) and (11), the rebroadcasting signal considered all ESs can be expressed as

(12) $$R_{TR_{n}}\left(\omega \right)=\left[g_{n}A_{ng}\Gamma_{n}R_{n}^{*}+V_{n}\right]I$$

Thus, we can get $R_{TR}\left(\omega \right)={\left[R_{TR_{1}}\left(\omega \right),R_{TR_{2}}\left(\omega \right),\cdots ,R_{TR_{N}}\left(\omega \right)\right]}^{T}$, which is defined as a (N×1) vector representing TR-processed signal of array. According to Equation (7), and combine with Capon, the TR-Capon-DOA takes the form

(13) $$\left\{\begin{array}{c} \min_{W_{TR}}={W_{TR}}^{H}\left(\omega ,\theta \right)B_{TR}\left(\omega ,\theta \right)W_{TR}\left(\omega ,\theta \right)\\ s.t.{W_{TR}}^{H}\left(\omega ,\theta \right)a_{TR}\left(\omega ,\theta \right)=1 \end{array}\right.$$

in which $B_{TR}\left(\omega ,\theta \right)=E\left[R_{TR}\left(\omega \right)R_{TR}^{H}\left(\omega \right)\right]$, and $a_{TR}\left(\omega ,\theta \right)=a\left(\omega ,\theta \right)$ is the TR steering vector. The TR weight vector and TR power spectrum can be expressed respectively as

(14) $$W_{TR}\left(\omega ,\theta \right)=\frac{B_{TR}^{-1}\left(\omega ,\theta \right)a_{TR}\left(\omega ,\theta \right)}{a_{TR}^{H}\left(\omega ,\theta \right)B_{TR}^{-1}\left(\omega ,\theta \right)a_{TR}\left(\omega ,\theta \right)}$$

(15) $$P_{TR}\left(\omega ,\theta \right)=\frac{1}{a_{TR}^{H}\left(\omega ,\theta \right)B_{TR}^{-1}\left(\omega ,\theta \right)a_{TR}\left(\omega ,\theta \right)}$$

The values of DOAs are obtained from θ corresponding to *K* peaks of Equation (15).

#### 2.2.3. Suppressing Noise DOA Estimation Based on TR

**Figure 5:**
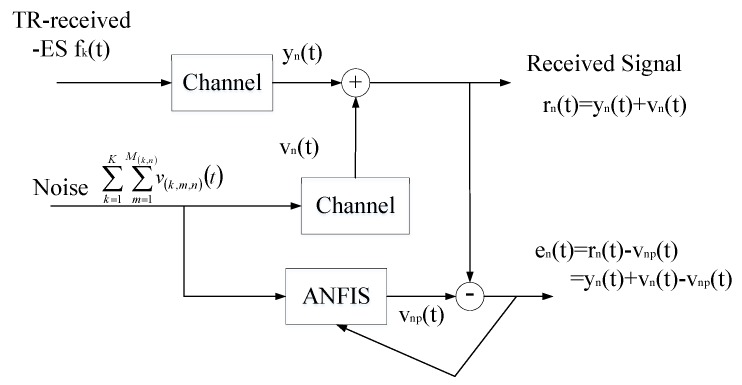
Schematic diagram for noise suppression using ANFIS

In order to improve the performance of DOA estimation further, the noise need to be suppressed, which is also the purpose of this paper. Actually, there are two kinds of noise as shown in Equation (11). Therein, the noise2 part is background noise without any distortion following the Gaussian distribution. This kind of noise can be measured by pre-test. The noise 1 and clutter2 parts put together as the other kind of noise, which is distorted because of rebroadcasting. Besides, this kind of noise is random and difficult to find its property because of high level of uncertainty. Moreover, it is shown that the spectrum of noise overlaps that of useful signal substantially, which invalidates the common filtering techniques to cancel noise. Thus, in this section, adaptive noise cancellation is used to suppress this kind of noise. In order to estimate the received noise, which is different from the original noise because of noise distortion happening during the retransmission process, a clean version of noise that is independent and uncorrelated of the useful signal need to be picked up. However, the noise cannot be accessed directly since it is an additive component of the overall received signal. Fortunately, this distorted noise can be recovered with the adaptive fuzzy system trained with a neural network called ANFIS here as shown in Figure 5. ANFIS model combines the fuzzy system and neural network capabilities. This neuro-fuzzy system is a system that uses a learning algorithm derived from or inspired by neural network theories that determine rules created by fuzzy system with analyzing samples. The specific processes are given below.

This ANFIS has two inputs: the noise and the error signal $e_{n}\left(t\right)$. Therein, the measurable background noise can be used as the initial value of noise. Although it is not accuracy enough, it will be corrected during the training process and replaced with $v_{np}\left(t\right)$ after going through ANFIS, that is, $v_{np}\left(t\right)$ will be used as the next initial noise. The error signal $e_{n}\left(t\right)$ represents the difference between received signal $r_{n}\left(t\right)$ and pure received signal from ES $y_{n}\left(t\right)$. ANFIS accepts $e_{n}\left(t\right)$ to control and adjust the weights that decide the output of ANFIS, here is denoted as $v_{np}\left(t\right)$. Moreover, the output of ANFIS needs to be adjusted approximately equal to the distorted noise $v_{n}\left(t\right)$, in order to retrieve useful received signal–ES $y_{n}\left(t\right)$ after several circles.

**Figure 6:**
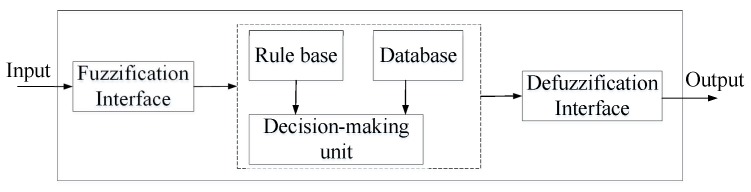
Structure of fuzzy system.

Mathematically, the received signal can be expressed as $r_{n}\left(t\right)=y_{n}\left(t\right)+v_{n}\left(t\right)=y_{n}\left(t\right)+\sum_{k=1}^{K}\sum_{m=1}^{M_{\left(k,n\right)}}h_{\left(k,m,n\right)}\left(v_{\left(k,m,n\right)}\left(t\right),v_{\left(k,m,n\right)}\left(t-1\right),v_{\left(k,m,n\right)}\left(t-2\right),\cdots \right)$, where the function $h_{\left(k,m,n\right)}\left(\cdot \right)$ represents the non-linear operation corresponding to the *m*th path from ES *k* to antenna *n* where the noise $v_{\left(k,m,n\right)}\left(t\right)$ goes through. If $h_{\left(k,m,n\right)}\left(\cdot \right)$ was known exactly, it would be easy to recover the TR-resubmitting signal from ES *k* by subtracting $v_{n}\left(t\right)$ from $r_{n}\left(t\right)$ directly, because of the measurable background AWGN $v_{\left(k,m,n\right)}\left(t\right)$. However, $h_{\left(k,m,n\right)}\left(\cdot \right)$ is usually unknown in advance and could be time varying due to changes in the environment. Thereupon, ANFIS is adopted here to solve this problem. The ANFIS architecture can identify the near optimal membership functions of fuzzy systems in order to achieve the desired output of the whole noise suppression system, here is $e_{n}\left(t\right)$. More specifically, the learning rule of the neural network tries to minimize the error ${\left[e_{n}\left(t\right)\right]}^{2}={\left[r_{n}\left(t\right)-v_{np}\left(t\right)\right]}^{2}={\left[y_{n}\left(t\right)+v_{n}\left(t\right)-v_{np}\left(t\right)\right]}^{2}=[y_{n}\left(t\right)+v_{n}\left(t\right)-\sum_{k=1}^{K}\sum_{m=1}^{M_{\left(k,n\right)}}x_{\left(k,m,n\right)}\left(v_{\left(k,m,n\right)}\left(t\right),v_{\left(k,m,n\right)}\left(t-1\right),v_{\left(k,m,n\right)}\left(t-2\right),\cdots \right)$, where the function $x_{\left(k,m,n\right)}\left(\cdot \right)$ is the non-linear function implemented by the fuzzy system in ANFIS. The structure of fuzzy system is shown in Figure 6. This fuzzy system uses fuzzy theory and membership function. Firstly, compare the input variables with the membership functions of desired signal $v_{np}\left(t\right)$ on the premise part. Therein, the membership functions of the fuzzy sets used in fuzzy rules are defined in the database block, such as trapezoidal, or triangular or bell-shaped membership functions. Here uses bell-shaped membership whose expression is $\mu_{A_{i}}=\frac{1}{1+\left[{\left(\frac{v_{np}\left(t\right)-c_{i}}{a_{i}}\right)}^{2}\right]b_{i}}$, therein, $A_{i}$ is the linguistic label (small, large, etc.), $\left\{a_{i},b_{i},c_{i}\right\}$ is the parameter set. As the values of these parameters change, the bell-shaped functions vary accordingly, therefore, exhibiting various forms of membership functions on linguistic label $A_{i}$. The membership function specifies the degree to which the given *v* satisfies the quantifier $A_{i}$. Moreover, the membership values can also be obtained through a specific T-norm operator which is usually multiplication or min. The return value is the degree of match with decisions. Secondly, combine with membership values, get weight (obtained in decision-making unit) of each fuzzy if-then rule (contained in rule base) as $W_{i}=\mu_{A_{i}}\left(v_{np}\left(t\right)\right)\times \mu_{B_{i}}\left(e_{n}\left(t\right)\right)$, where $B_{i}$ is another linguistic label, so that the qualified fuzzy or crisp consequent of each rule is generated after several loops. Namely, the final output of this fuzzy system is the weighted average of all the rule outputs computed as $v_{np}\left(t\right)=\frac{\sum_{i=1}^{I}W_{i}v_{np_{i}}\left(t\right)}{\sum_{i=1}^{I}W_{i}}$, therein, *I* and *W* denote the total number of training and weight respectively. At last, aggregate the qualified consequents to produce a crisp output, which is relatively accurate TR-resubmitting signal of ES *k*, namely $e_{n}\left(t\right)\approx y_{n}\left(t\right)$. Furthermore, several loops will be taken if necessary.

In summary, the steps of ANFIS [46] are shown as follows:

(a). Compare the input variables with the membership functions on the premise part, so that the membership values or compatibility measures of each decision can be obtained. This step is always called fuzzification. This step needs a fuzzification interface block which transforms the crisp inputs into degrees of match with decisions.
(b). Combine the membership values on the premise part to get weight of each fuzzy if-then rule. Therein, the membership values can be obtained through a specific T-norm operator which is usually multiplication or min. Then, generate the qualified fuzzy or crisp consequent of each fuzzy if-then rule depending on weight.This step need three functional blocks-a rule base, a database, a decision-making unit. Therein, a rule base contains a plenty of fuzzy if-then rules; a database defines the membership functions of the fuzzy sets used in fuzzy rules; and a decision-making unit performs the inference operations upon the rules and gets the weight. Usually, the rule base and the database are jointly referred to as the knowledge base.
(c). Aggregate the qualified consequents to develop a crisp output. This step is called defuzzification which need a defuzzification interface block. This block transforms the fuzzy results of the inference into a crisp output.

After processing the received signal $r_{n}\left(t\right)$ by employing ANFIS, we use $e_{n}\left(t\right)$ as received signal, and then estimate the DOA with the help of TR-Capon-DOA algorithm discussed above.

According to the analysis above, the TR-NS-Capon-DOA can be obtained. Firstly, denote $C\left(\omega \right)={\left[{Er}_{1}\left(\omega \right),{Er}_{2}\left(\omega \right)\cdots {Er}_{N}\left(\omega \right)\right]}^{T}$, which is a (N×1) vector, therein, ${Er}_{n}\left(\omega \right)$ is the Fourier transform (presentation in frequency domain) of $e_{n}\left(t\right)$. The following process is similar to Section 2.2.2. As a result, the weight vector and power spectrum can be got respectively as

(16) $$W_{C}\left(\omega ,\theta \right)=\frac{B_{C}^{-1}\left(\omega ,\theta \right)a_{C}\left(\omega ,\theta \right)}{a_{C}^{H}\left(\omega ,\theta \right)B_{C}^{-1}\left(\omega ,\theta \right)a_{C}\left(\omega ,\theta \right)}$$

(17) $$P_{C}\left(\omega ,\theta \right)=\frac{1}{a_{C}^{H}\left(\omega ,\theta \right)B_{C}^{-1}\left(\omega ,\theta \right)a_{C}\left(\omega ,\theta \right)}$$

in which $B_{c}\left(\omega ,\theta \right)=E\left[C\left(\omega \right)C^{H}\left(\omega \right)\right]$, and $a_{C}\left(\omega ,\theta \right)=a\left(\omega ,\theta \right)$ is the steering vector. The values of DOAs are acquired from θ corresponding to *K* peaks of Equation (17).

Compared with some conventional methods, such as signal classification and decomposition of operator theories, the proposed algorithm does not need to construct the multistatic data matrix and analyze the eigenvalue and eigenvectors of multistatic data matrix in the signal process stage. Furthermore, just only consider Fourier transform which is also needed in the conventional theories. Therefore, the proposed theory is less algorithm complexity.

#### 2.2.4. DOA Estimation Performance Based on RMSE and CRLB

This section introduces the root mean square error (RMSE) and Cramér-Rao lower bound (CRLB) to evaluate the performance of DOA estimation. Therein, the average RMSE considers all snapshots, and its formula deduced from the *k*th ES is defined as

(18) $$RMSE_{k}=\sqrt{\frac{1}{Q}\sum_{n_{k_{t}}=1}^{Q}{\left|\theta_{n_{t_{k}}}-{\hat{\theta }}_{n_{t_{k}}}\right|}^{2}}$$

where $\theta_{n_{t_{k}}}$ is the true DOA value and ${\hat{\theta }}_{n_{t_{k}}}$ is the estimated DOA value at the $n_{t_{k}}$ snapshot.

The CRLB provides a lower bound on the covariance matrix of any unbiased estimator [47,48]. Here, we assume that $\hat{\theta }=\left[{\hat{\theta }}_{1},{\hat{\theta }}_{2},\cdots ,{\hat{\theta }}_{K}\right]$ is the estimated value of directional vector $\theta =[\theta_{1},\theta_{2},\cdots ,\theta_{K}]$.

Additionally, the precision can also be judged by the Fisher information matrix (FIM) $I(\theta_{k})$ and stochastic CRLB of directional vector $\theta_{k}$, which is the inversion of the FIM. According to [24], the FIM is a function of $B\left(\omega ,\theta \right)=E[R\left(\omega \right)R^{H}\left(\omega \right)]$, $B_{TR}\left(\omega ,\theta \right)=E\left[R_{TR}\left(\omega \right)R_{TR}^{H}\left(\omega \right)\right]$, and $B_{c}\left(\omega ,\theta \right)=E\left[C\left(\omega \right)C^{H}\left(\omega \right)\right]$ for corresponding methods, and its general expression is

(19) $$\begin{array}{c} \mathrm{FIM}=QTr{\left[\frac{\partial R}{\partial \xi_{i}}\right]}^{H}{\left(B^{T}⊗B\right)}^{-1}\left[\frac{\partial R}{\partial \xi_{j}}\right] \end{array}$$

where $\xi_{i}$ and $\xi_{j}$ are the *i* th and *j* th elements of $\xi =[\theta ,\rho ,\sigma^{2}]$. Let $\Phi_{n}$ be a $\left(\sum_{k=1}^{K}M_{\left(k,n\right)}\times 1\right)$ matrix made from the diagonal elements of $\left[\Gamma_{n}⊗\Gamma_{n}^{H}\right]$ which is the $\left(\sum_{k=1}^{K}M_{\left(k,n\right)}\times \sum_{k=1}^{K}M_{\left(k,n\right)}\right)$ diagonal matrix [24,49], and $\rho_{k}=\left[\Phi_{1}\left[\sum_{z=1}^{k-1}M_{\left(z,1\right)}+1,\cdots ,\sum_{z=1}^{k-1}M_{\left(z,1\right)}+M_{\left(k,1\right)}\right],\Phi_{2}\left[\sum_{z=1}^{k-1}M_{\left(z,2\right)}+1,\cdots ,\sum_{z=1}^{k-1}M_{\left(z,2\right)}+M_{\left(k,2\right)}\right],\cdots ,\Phi_{N}\left[\sum_{z=1}^{k-1}M_{\left(z,N\right)}+1,\cdots ,\sum_{z=1}^{k-1}M_{\left(z,N\right)}+M_{\left(k,N\right)}\right]\right]$ is a $\left(\sum_{n=1}^{N}M_{\left(k,n\right)}\times 1\right)$ matrix. Then, we can get $\rho =[\rho_{1},\rho_{2},\cdots ,\rho_{K}]$. $\sigma^{2}=\left[\sigma_{1}^{2},\sigma_{2}^{2},\cdots ,\sigma_{K}^{2}\right]$, therein, $\sigma_{k}^{2}=\left[\sigma_{\left(k,1,n\right)}^{2},\sigma_{\left(k,2,n\right)}^{2},\cdots ,\sigma_{\left(k,M_{\left(k,N\right)},N\right)}^{2}\right]$ is a $\left(\sum_{n=1}^{N}M_{\left(k,n\right)}\times 1\right)$ matrix. When the number of ES exceeds the number of antenna elements, this FIM is singular (proof see [24]), resulting in the conventional stochastic CRLB inapplicable. Thus, transform the FIM into a virtual array-based form, which keeps nonsingular within a much broader range of conditions and overcomes the model mismatch issue of the conventional stochastic CRLB. In particular, this form presents a relative lower bound for the estimation error even when the number of ES is larger than the number of antenna elements. In our case, the representations can be expressed as

(20) $$\left\{\begin{array}{c} {\mathrm{FIM}}_{Capon}=Q{\left[\frac{\partial R}{\partial \xi }\right]}^{H}{\left(B^{T}⊗B\right)}^{-1}\left[\frac{\partial R}{\partial \xi }\right]\\ {\mathrm{FIM}}_{TR-Capon-DOA}=Q{\left[\frac{\partial R_{TR}}{\partial \xi }\right]}^{H}{\left(B_{TR}^{T}⊗B_{TR}\right)}^{-1}\left[\frac{\partial R_{TR}}{\partial \xi }\right]\\ {\mathrm{FIM}}_{TR-NS-Capon-DOA}=Q{\left[\frac{\partial C}{\partial \xi }\right]}^{H}{\left(B_{C}^{T}⊗B_{C}\right)}^{-1}\left[\frac{\partial C}{\partial \xi }\right] \end{array}\right.$$

where

(21) $$\left\{\begin{array}{c} \frac{\partial R}{\partial \xi }=\left[\frac{\partial R}{\partial \theta_{1}},\cdots ,\frac{\partial R}{\partial \theta_{K}},\frac{\partial R}{\partial \rho_{1}},\cdots ,\frac{\partial R}{\partial \rho_{K}},\frac{\partial R}{\partial \sigma_{1}^{2}},\cdots ,\frac{\partial R}{\partial \sigma_{K}^{2}}\right]\\ \frac{\partial R_{TR}}{\partial \xi }=\left[\frac{\partial R_{TR}}{\partial \theta_{1}},\cdots ,\frac{\partial R_{TR}}{\partial \theta_{K}},\frac{\partial R_{TR}}{\partial \rho_{1}},\cdots ,\frac{\partial R_{TR}}{\partial \rho_{K}},\frac{\partial R_{TR}}{\partial \sigma_{1}^{2}},\cdots ,\frac{\partial R_{TR}}{\partial \sigma_{K}^{2}}\right]\\ \frac{\partial C}{\partial \xi }=\left[\frac{\partial C}{\partial \theta_{1}},\cdots ,\frac{\partial C}{\partial \theta_{K}},\frac{\partial C}{\partial \rho_{1}},\cdots ,\frac{\partial C}{\partial \rho_{K}},\frac{\partial C}{\partial \sigma_{1}^{2}},\cdots ,\frac{\partial C}{\partial \sigma_{K}^{2}}\right] \end{array}\right.$$

Thus, we can obtain the CRLB for the *k*-th ES as

(22) $${\left(CRLB\right)}_{\theta_{k}}={\left[{\mathrm{FIM}}^{-1}\right]}_{\left(k,k\right)}$$

## 3. Numerical Experiment

**Figure 7:**
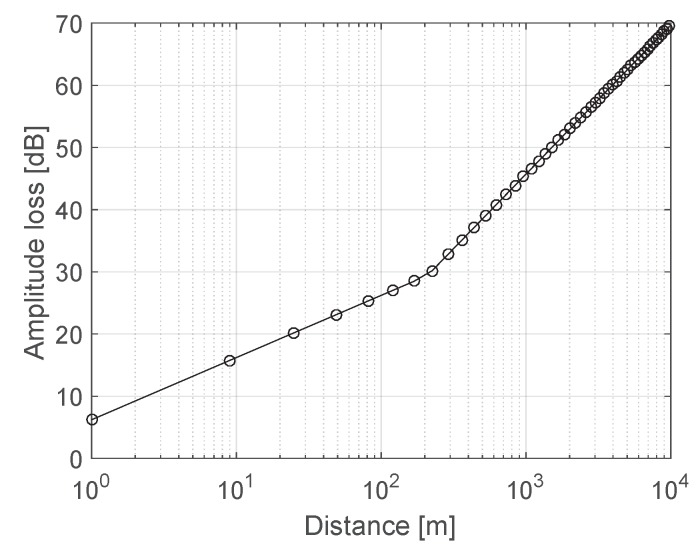
Amplitude loss of signal versus distance between ES and antenna.

In this section, the performance of DOA estimation based on the proposed TR-NS-Capon-DOA algorithm, TR-Capon-DOA method and OCA configuration are investigated. The results are compared with those obtained by the conventional Capon theory, ULA, CA, NA and MRA structures. Assuming array contains N=13 antennas, ESs may be linear frequency modulation (LFM) signal and nonlinear frequency modulation (NLFM) signal or other kinds of signal, these signals are not able to be known in advance. Here, we take LFM and NLFM for example to prove that the ability of the proposed methods in obtaining the DOA of ES is independent of the type of ES. Therein, the representation of LFM is $f\left(t\right)=e^{j(2\pi f_{c}t+k_{p}\pi t^{2})}$, $k_{p}$ is chirp slope, $f_{c}=0.1\;GHz$ is the carrier frequency, and the representation of NLFM is $f\left(t\right)=e^{j\varphi (t)}$, $\varphi \left(t\right)=2\pi \left(f+f_{c}\right)t$, $f_{c}=0.1\;GHz$ is also the carrier frequency, and the total number of snapshots is 9000. According to log-normal shadowing model, the amplitude loss of signal versus distance is shown in Figure 7. Here, assuming the horizontal distance between ES and the first antenna element equals to dh=1 km. Thus, the distance between ES and antenna 1 used as reference element equals dh/sin(DOA).

### 3.1. Multipath DOA Estimation with ULA

**Figure 8:**
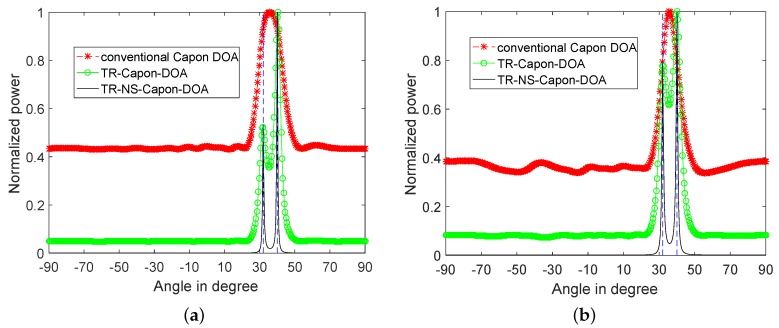
2-path normalized power spectra versus angles using conventional Capon DOA, TR-Capon-DOA and TR-NS-Capon-DOA algorithms under the condition of SNR = −15 dB and ULA, (**a**) LFM (**b**) NLFM.

According to 1D-FDTD, reference time delay are $\tau_{ref}=5.19\;\mu$s and 6.3μs for DOA = $40^{\circ }$ and $32^{\circ }$ respectively. When SNR is −15 dB, the normalized power spectra produced by conventional Capon DOA, TR-Capon-DOA and TR-NS-Capon-DOA algorithms are plotted in Figure 8, where for reference two vertical dotted blue lines at $40^{\circ }$ and $32^{\circ }$ are drawn corresponding to the actual DOA values. It is shown that there is only one peak at DOA = $36^{\circ }$ presented in the conventional Capon DOA power spectrum under the conditions of LFM and NLFM signals used as ESs, which is in the middle of DOA = $32^{\circ }$ and $40^{\circ }$ with an error of $4^{\circ }$. However, the TR-Capon-DOA algorithm produces a better result, there are two peaks at DOA = $31.5^{\circ }$ and $40.5^{\circ }$ with an error of $0.5^{\circ }$ in the LFM case, and two peaks at DOA = $31.95^{\circ }$ and $40.05^{\circ }$ with an error of $0.05^{\circ }$ in the NLFM case. Process the TR signal through ANFIS, the more precise results can be obtained using TR-NS-Capon-DOA algorithm. There are two peaks at DOA = $31.95^{\circ }$ and $40.05^{\circ }$ with an error of $0.05^{\circ }$ in both cases. Although the same accuracy can be obtained by using TR-Capon-DOA and TR-NS-Capon-DOA algorithms in NLFM case, the sidelobes can be suppressed effectively and high resolution can be obtained obviously by TR-NS-Capon-DOA methodology. Furthermore, when it comes to passive array, the estimation error of DOA can be reduced and the estimation resolution of DOA can be improved by the proposed TR-Capon-DOA and TR-NS-Capon-DOA methods compared with the conventional Capon method.

**Figure 9:**
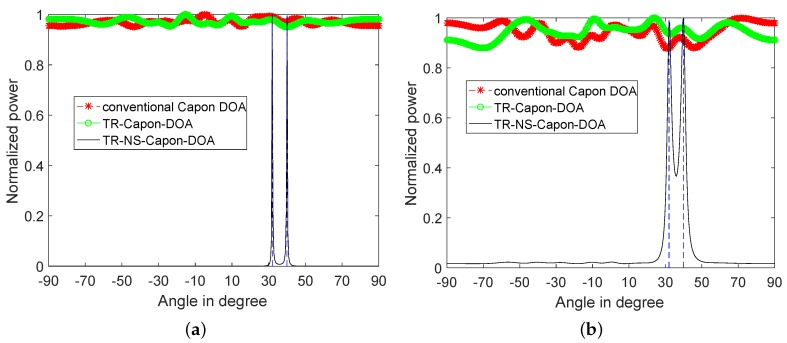
2-path normalized power spectra versus angles using conventional Capon DOA, TR-Capon-DOA and TR-NS-Capon-DOA algorithms under the condition of SNR = −100 dB and ULA, (**a**) LFM (**b**) NLFM.

For investigating the effect of noise on DOA estimation, and the performance of the proposed method on suppressing noise, the condition of SNR = −100 dB is analyzed and the results are shown in Figure 9. It can be seen that the DOAs are unable to be estimated completely except with the help of TR-NS-Capon-DOA algorithm. Furthermore, the DOA = $31.95^{\circ }$ and $40.05^{\circ }$ with an error of $0.05^{\circ }$ are achieved. In short, the proposed TR-NS-Capon-DOA algorithm can suppress the noise effectively and achieve a high resolution and accuracy.

**Figure 10:**
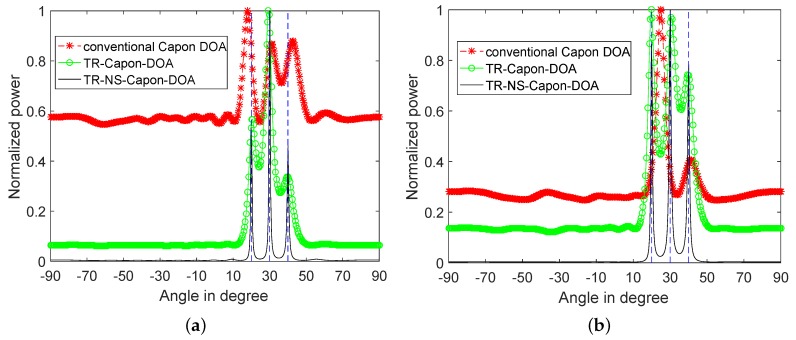
3-path normalized power spectra versus angles for conventional Capon DOA, TR-Capon-DOA and TR-NS-Capon-DOA algorithms under the condition SNR = −15 dB and ULA, (**a**) LFM (**b**) NLFM.

Furthermore, for researching the performance of the proposed method in the cases of more multipath, three paths are introduced for DOA = $40^{\circ }$, $30^{\circ }$, $20^{\circ }$ with the reference time delay $\tau_{ref}=5.19\;\mu$s, 6.6667μs and 9.7460μs respectively. Other parameters are set as the same as the 2-path case. Again, as Figure 10 shows, there are three peaks at DOA = $18^{\circ }$, $31.15^{\circ }$ and $43.2^{\circ }$ presented in the conventional Capon DOA power spectrum with errors of $2^{\circ }$, $1.15^{\circ }$ and $3.2^{\circ }$ respectively in the LFM case, and only two peaks at DOA = $24.75^{\circ }$, and $41.4^{\circ }$ with errors of $4.75^{\circ }$, and $1.4^{\circ }$ in the NLFM case. It is also indicated that the TR-Capon-DOA algorithm is more accurate with narrower three peaks at DOA = $20.7^{\circ }$, $29.25^{\circ }$ and $39.6^{\circ }$, which has $0.7^{\circ }$, $0.75^{\circ }$ and $0.4^{\circ }$ errors respectively in the LFM case, and there are also three peaks at DOA = $19.8^{\circ }$, $30.6^{\circ }$ and $39.6^{\circ }$ with errors of $0.2^{\circ }$, $0.6^{\circ }$ and $0.4^{\circ }$ respectively in the NLFM case. Process the TR signal through ANFIS, the more precise results can be obtained by TR-NS-Capon-DOA algorithm. There are three peaks at DOA = $19.8^{\circ }$, $30.15^{\circ }$ and $40.05^{\circ }$, whose errors are respectively $0.2^{\circ }$, $0.15^{\circ }$ and $0.05^{\circ }$ in the LFM case, and there are also three peaks at DOA = $19.8^{\circ }$, $30.15^{\circ }$ and $40.05^{\circ }$ with errors of $0.2^{\circ }$, $0.15^{\circ }$ and $0.05^{\circ }$ respectively in the NLFM case.

**Figure 11:**
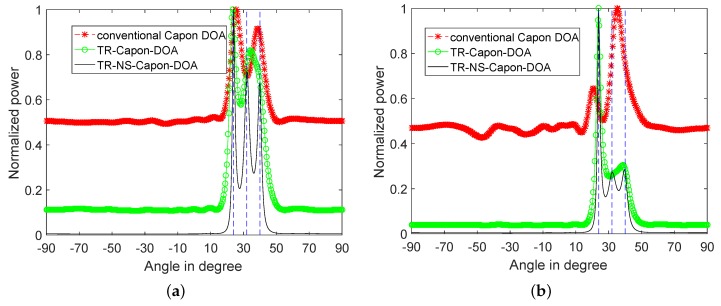
3 near paths’ normalized power spectra versus angles for conventional Capon DOA, TR-Capon-DOA and TR-NS-Capon-DOA algorithms under the condition of SNR = −15 dB and ULA, (**a**) LFM (**b**) NLFM.

Next, in order to investigate the performance of near DOA estimation. Repeat 3-path simulation under the condition of DOA = $40^{\circ }$, $32^{\circ }$ and $24^{\circ }$, and the corresponding time delay $\tau_{ref}=5.19\;\mu$s, 6.3μs and 8.21μs respectively. As shown in Figure 11, in both cases, three DOAs cannot be recognized by the conventional Capon algorithm because of insufficient precision, and TR-Capon-DOA algorithm because of the existed noise. Specifically, DOA = $25.65^{\circ }$ and $38.7^{\circ }$ can be obtained by the conventional Capon algorithm, DOA = $23.4^{\circ }$ and $34.2^{\circ }$ are got by TR-Capon-DOA algorithm in the LFM case, DOA = $20.7^{\circ }$ and $35.1^{\circ }$ can be accessed by the conventional Capon algorithm, and DOA = $23.85^{\circ }$ and $38.7^{\circ }$ are achieved by TR-Capon-DOA algorithm in the NLFM case. However, much better results can be obtained by the proposed TR-NS-Capon-DOA algorithm. Namely, DOA = $23.85^{\circ }$, $31.95^{\circ }$ and $40.05^{\circ }$ with errors of $0.15^{\circ }$, $0.05^{\circ }$ and $0.05^{\circ }$ in the LFM case, and DOA = $23.85^{\circ }$, $32.4^{\circ }$ and $39.6^{\circ }$ with errors of $0.15^{\circ }$, $0.4^{\circ }$ and $0.4^{\circ }$ in the NLFM case have already achieved.

From the analysis and results, we can conclude that the proposed TR-NS-Capon-DOA estimation algorithm has a superior performance compared with the conventional Capon, and even better than the proposed TR-Capon-DOA algorithm, whose performance is better than that of the conventional Capon. In addition, similar results were obtained with other numerical simulations under the conditions of different multipaths, DOAs, SNRs and ESs containing frequency shift keying (FSK) ES, general pulse ES, etc.

### 3.2. Multipath DOA Estimation with CA and Optimized CA

**Figure 12:**
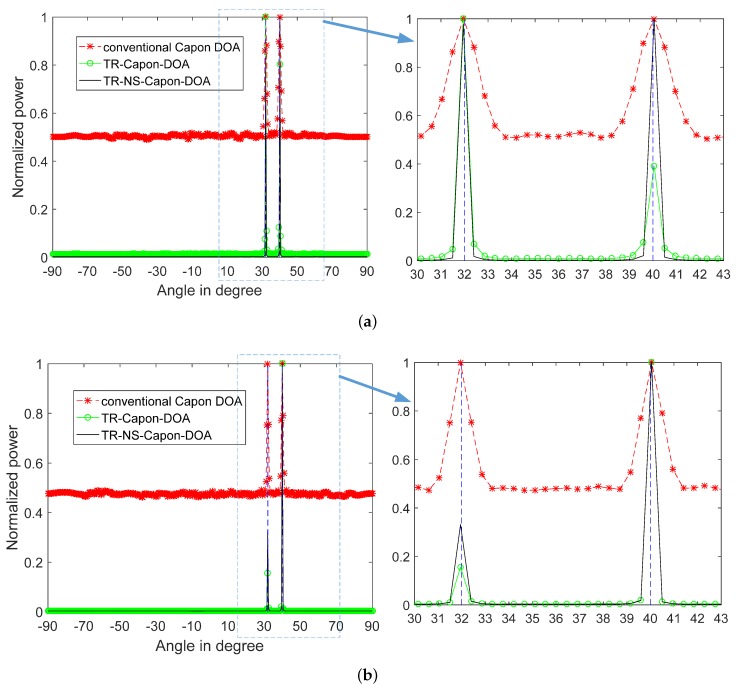
2-path normalized power spectra versus angles for conventional Capon DOA, TR-Capon-DOA and TR-NS-Capon-DOA algorithms under the condition of LFM used as ES, SNR = −15 dB and (**a**) conventional CA (**b**) Optimized CA.

**Figure 13:**
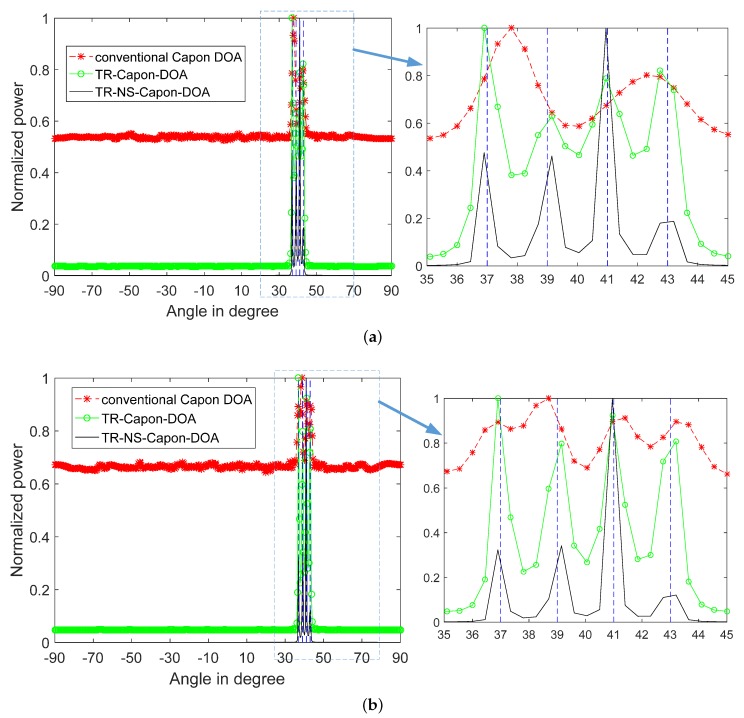
4-path normalized power spectra versus angles for conventional Capon DOA, TR-Capon-DOA and TR-NS-Capon-DOA algorithms under the condition of LFM used as ES, SNR = −15 dB and (**a**) conventional CA (**b**) Optimized CA.

For investigating the influence of CA and OCA arrangements on the resolution and accuracy for DOA estimation, the 2-path DOA estimation (DOA = $32^{\circ }$ and $40^{\circ }$) is researched for contrastive study. Take the LFM used as ES for example, the results are shown in Figure 12, and the results generated by other kinds of ESs including NLFM are similar. It can be seen that the extreme accurate and exceeding high resolution DOA can be obtained by using three DOA estimation algorithms in both CA and OCA cases. The results are much better than that produced by ULA. Moreover, hardly difference exists between these two results in Figure 12, because the accuracy limit value is achieved, namely, the accuracy is not able to be optimized further with the increasement of array aperture. However, if the number of paths increases, especially, the near paths, the superiority of OCA appears. As shown in Figure 13, 4 paths with DOA = $37^{\circ }$, $39^{\circ }$, $41^{\circ }$ and $43^{\circ }$, and the corresponding time delay $\tau_{ref}=4.9\;\mu$s, 5.1μs, 5.3μs and 5.5μs respectively. Under the condition of CA arrangement, using conventional Capon algorithm, only two peaks can be obtained at DOA = $37.8^{\circ }$ and $42.3^{\circ }$, while four peaks at DOA = $36.9^{\circ }$, $39.15^{\circ }$, $40.95^{\circ }$ and $42.75^{\circ }$ are able to be obtained by TR-Capon-DOA and four peaks at DOA = $36.9^{\circ }$, $39.15^{\circ }$, $40.95^{\circ }$ and $43.2^{\circ }$ can be accessed by TR-NS-Capon-DOA algorithm. It is also obvious that although the accuracy is almost the same with the use of TR-Capon-DOA and TR-NS-Capon-DOA estimations, the resolution got by TR-NS-Capon-DOA is higher than that obtained by TR-Capon-DOA algorithm. Otherwise, under the condition of OCA arrangement, because larger array aperture compared with CA arrangement is achieved and accuracy limit value is not reached, four peaks (DOA = $36.9^{\circ }$, $38.7^{\circ }$, $41.4^{\circ }$ and $43.2^{\circ }$ obtained by the conventional Capon method, $36.9^{\circ }$, $39.15^{\circ }$, $40.95^{\circ }$ and $43.2^{\circ }$ got by using TR-Capon-DOA theory, and $36.9^{\circ }$, $39.15^{\circ }$, $40.95^{\circ }$ and $42.75^{\circ }$ accessed by making use of TR-NS-Capon-DOA methodology) are all obtained by taking advantage of these three algorithms. Besides, the resolution based on TR is higher than that based on Capon. Moreover, compared with TR-Capon-DOA, the TR-NS-Capon DOA has a better resolution, since the small sidelobes are achieved. While, the accuracies obtained by these two TR methods are almost the same, because the accuracy limit value reaches. It is worth mention that the resolution and accuracy under the circumstance of OCA arrangement is higher than that in the case of conventional CA arrangement. In a word, the proposed OCA can improve the resolution and accuracy of DOA estimation greatly compared with conventional CA, especially, ULA.

### 3.3. Multipath DOA Estimation with OCA, MRA and NA

**Figure 14:**
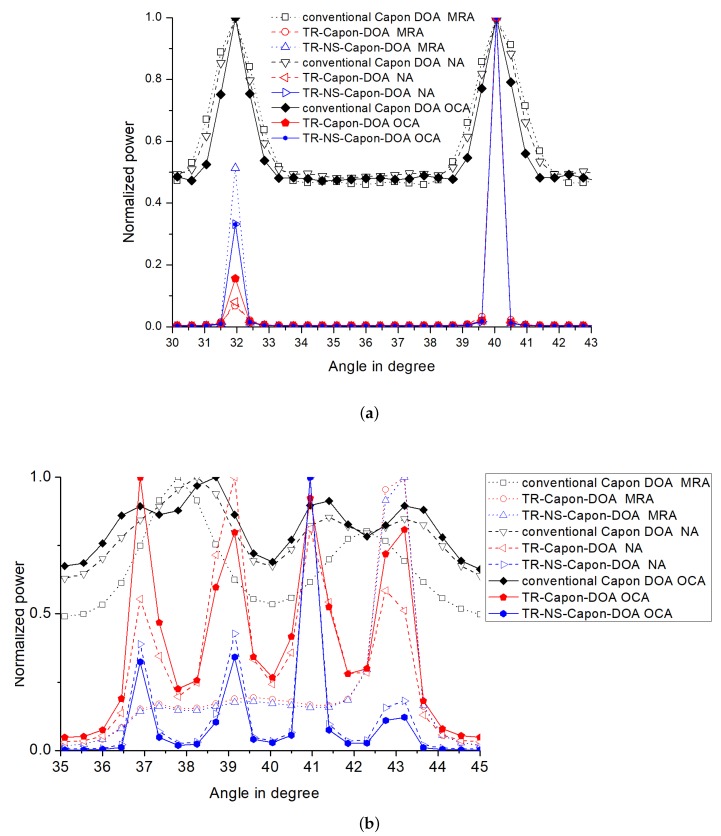
Normalized power spectra versus angles for conventional Capon DOA, TR-Capon-DOA and TR-NS-Capon-DOA algorithms with the configurations of MRA, NA and OCA under the condition of LFM used as ES, SNR = −15 dB and (**a**) 2-path (**b**) 4-path.

In order to prove the superiority of the proposed OCA on DOA estimation further, the same DOA estimation process is repeated with the configuration of MRA and NA. The results are as Figure 14 shown. It can be seen from Figure 14a that in 2-path environment, by using the conventional Capon algorithm, the same resolution is obtained in the MRA, NA and OCA cases. While, the sidelobes obtained in the proposed OCA case are smaller than those in NA case which are smaller than those in MRA case. The reason is that compared with MRA, the DOF in OCA case is higher, and when it comes to NA, the spacing between NA elements is small, which raises the mutual coupling effects [50], and affect the performance of DOA estimation negatively [51]. Moreover, by taking advantage of TR-Capon-DOA and TR-NS-Capon-DOA algorithms, the sidelobes in all three cases are further narrowed. Because accuracy limit value is achieved, the same resolution and sidelobe are obtained in all three cases.

In contrast, in the 4-path environment shown in Figure 14b, the resolution and sidelobes are different in these three cases, because of unreachable accuracy limit value. Specifically, they make use of conventional Capon DOA algorithm, the four peaks of normalized power corresponding to actual DOA values cannot be distinguished in MRA and NA cases, especially in MRA case. The results will be better by TR-Capon-DOA and TR-NS-Capon-DOA algorithms except in MRA case. Only one peak at DOA = $43.2^{\circ }$ can be obtained and other three DOAs are lost in MRA case. On the contrary, four DOAs can be recognized in both NA and OCA cases. More specifically, from the view of algorithm, with the same array arrangement, and compared to the TR-Capon-DOA, TR-NS-Capon-DOA obtains the same resolution because of the reached accuracy limit value, and narrower sidelobes due to the noise suppression. Besides, from the view of array arrangement, with the same algorithm, compared to NA, the proposed OCA achieves a higher resolution and narrower sidelobes. The reason is the same as the analysis in 2-path environment. As a result, the OCA and TR-NS-Capon-DOA proposed in this paper has a better performance on DOA estimation.

**Figure 15:**
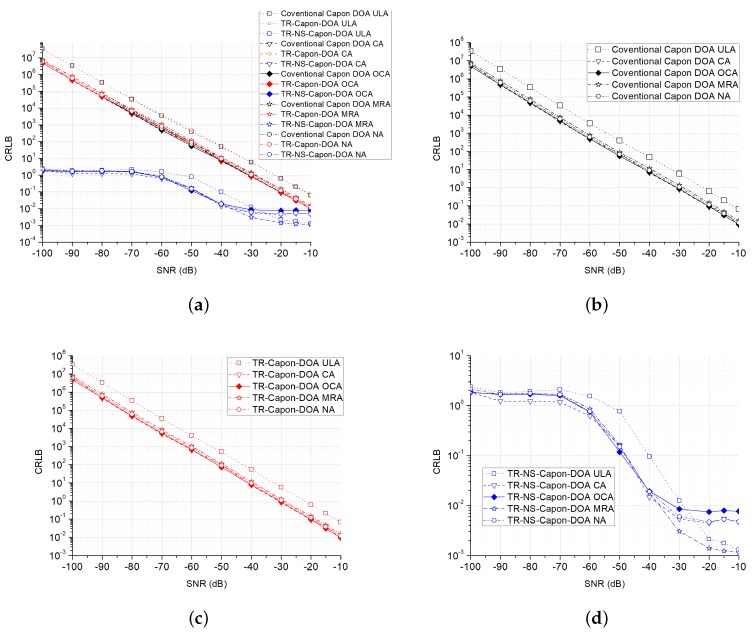
CRLB versus SNR for conventional Capon DOA, TR-Capon-DOA and TR-NS-Capon-DOA algorithms with the configurations of ULA, CA, OCA MRA and NA under the condition of LFM used as ES and DOA = $32^{\circ }$, (**a**) all methods and configurations; (**b**) the conventional method and all configurations; (**c**) the TR-Capon-DOA method and all configurations; (**d**) the TR-NS-Capon-DOA method and all configurations.

**Figure 16:**
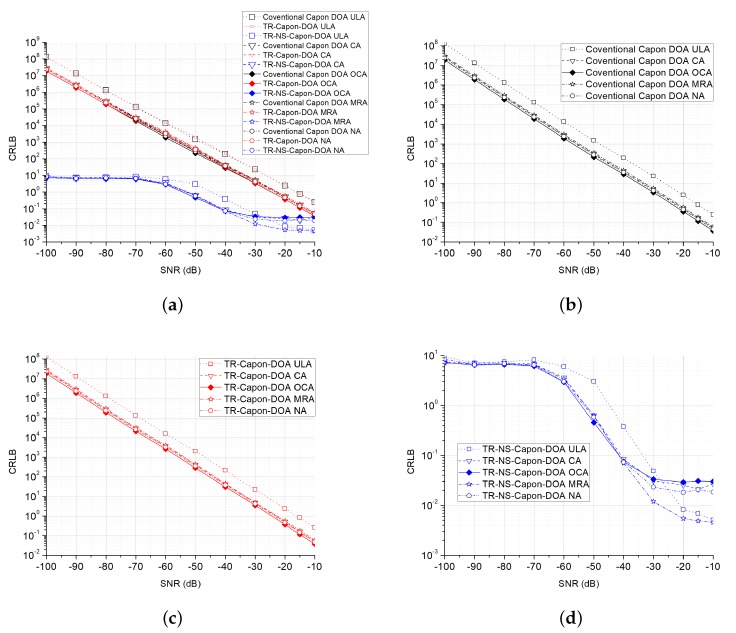
CRLB versus SNR for conventional Capon DOA, TR-Capon-DOA and TR-NS-Capon-DOA algorithms with the configurations of ULA, CA, OCA MRA and NA under the condition of LFM used as ES and DOA = $40^{\circ }$, (**a**) all methods and configurations; (**b**) the conventional method and all configurations; (**c**) the TR-Capon-DOA method and all configurations; (**d**) the TR-NS-Capon-DOA method and all configurations.

Furthermore, the CRLB and RMSE are also analyzed. One-hundred Monte Carlo trails are executed. Take the case of 2-path (DOA = $32^{\circ }$ and $40^{\circ }$) whose scenario is discussed above for example, the other cases with different paths are similar. In Figure 15 and Figure 16, at very low SNR condition, the proposed TR-NS-Capon-DOA estimation algorithm has a lower CRLB compared with the other two methods because of good noise suppression property. Different array configurations have the similar CRLB with the same algorithm. In addition, these CRLBs decrease with the increasing of SNR.

**Figure 17:**
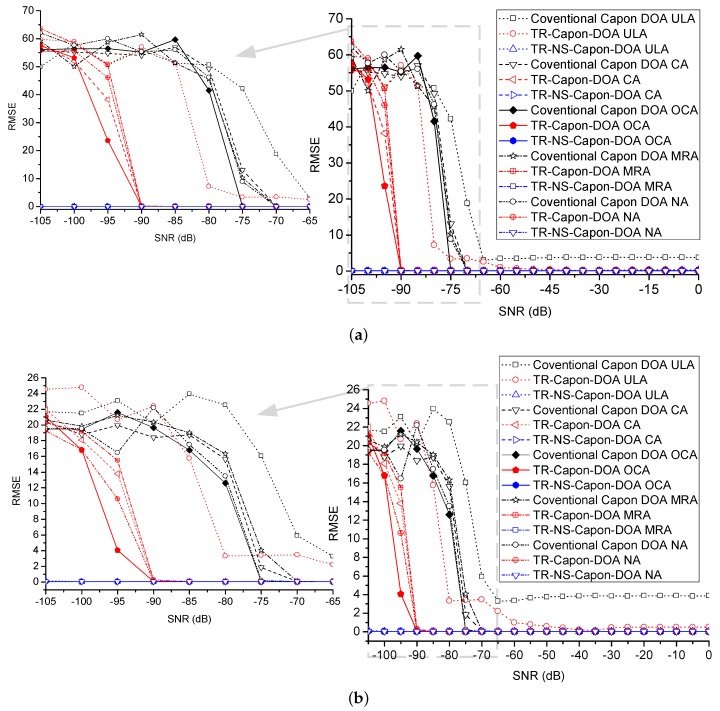
RMSE versus SNR for conventional Capon DOA, TR-Capon-DOA and TR-NS-Capon-DOA algorithms with the configurations of ULA, CA, OCA MRA and NA under the condition of LFM used as ES (**a**) DOA = $32^{\circ }$ (**b**) DOA = $40^{\circ }$.

RMSE is demonstrated in Figure 17. Compared with the conventional Capon and TR-Capon DOA methods, the proposed OCA configuration provides a faster convergence to the minimum. Furthermore, the TR-NS-Capon-DOA algorithm has a better performance compared with the other two methods because of excellent noise suppression. Moreover, by taking advantage of same array configuration, the performance of TR-NS-Capon-DOA algorithm is superior to that of TR-Capon-DOA algorithm, which is better than that of conventional Capon DOA theory. As a result, from the view of array configuration, the obtained performance ranges from good to bad as OCA, NA, MA, CA and ULA; from the view of algorithm, the obtained performance ranges from good to bad as TR-NS-Capon-DOA, TR-Capon-DOA and conventional Capon DOA.

**Figure 18:**
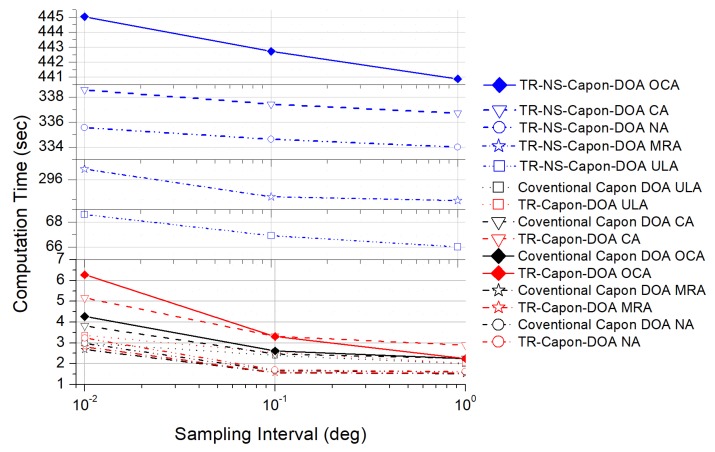
Computation time comparison with different sampling interval.

In the last example, we add the analysis of the computational complexity of the proposed method against the others. Namely, we compare the computational complexity measured by the computation time for 100 Monte Carlo trails on an Intel Core i7-7500U CPU, 8G RAM laptop, where the sampling/searching interval is varied. The result is shown in Figure 18. The computational complexities of these three algorithms all decrease when the sampling interval increases. This is because the pre-defined sampling interval increase the computational cost when solving the corresponding optimization problem. Additionally, during the signal processing, the TR-Capon-DOA algorithm needs to reverse the received signal in time domain firstly, then take the Capon operation on the time-reversed signal. Thus, the TR-Capon-DOA algorithm is a little more complex than the conventional Capon DOA algorithm. The difference value of computation time is between 0.18 s to 2.8 s, and the computation time of TR-Capon-DOA algorithm is just several seconds. Besides, the difference between TR-NS-Capon-DOA algorithm and TR-Capon-DOA algorithm is that the resubmitted distorted noise and channel noise appearing in the resubmitting stage needed to be trained and suppressed in TR-NS-Capon-DOA algorithm. However, this trained process can be executed during the pre-test stage before the DOA estimation stage. Because the resubmitted distorted noise and channel noise do not change with the unchanged channel and background noise. The distorted noise base (DNB) can be built after enough trails during the pre-test stage. Then, during the DOA estimation stage, the resubmitted distorted noise can be recognized very quickly through being compared with the data in DNB, only in the order of millisecond according to the sample size of DNB. Thus, the computation time of TR-NS-Capon-DOA algorithm is almost the same as that of TR-Capon-DOA algorithm. The efficiency of TR-NS-Capon-DOA algorithm is also good. Moreover, the computation time of TR-NS-Capon-DOA algorithm in Figure 18 includes the one -time trained time through ANFIS, which can be removed by pre-test.

## 4. Conclusions

For the sake of locating unknown active target using passive array in a multipath environment, this paper proposes a high resolution and accuracy DOA estimation algorithm with the property of noise suppression based on TR, Capon and OCA, gives analytical expressions, provides numerical experiments and analyzes the performance. The TR-NS-Capon-DOA estimation algorithm has a higher resolution, sharper peaks, narrower sidelobes and smaller errors compared with the conventional Capon algorithm whose performance is even worse than that of TR-Capon-DOA estimator. It shows that TR is an adaptive beamforming and channel matching technology, and can be used to enhance the accuracy and resolution of DOA estimation. 

## Figures and Tables

**Table 1:** Abbreviation used in the paper.

Abbreviation	Full Writting
ANFIS	Adaptive neuro-fuzzy interference system
CA	Coprime array
CRLB	Cramér-Rao lower band
DCA	Difference coprime array
DNB	Distorted noise base
DOA	Direction of arrival
DOF	Degree of freedom
ES	Emiiter signal
OCA	Optimized coprime array
RMSE	Root mean square error
SNR	Signal-to-noise ratio
TR	Time reversal
TR-Capon-DOA	Direction of arrival estimation based on time reversal and Capon
TR-DOA	Direction of arrival estimation based on time reversal
TR-NS-Capon-DOA	Direction of arrival estimation based on time reversal and Capon with the property of noise suppression
ULA	Uniform linear array
ULSA	Uniform linear subarray

**Table 2:** Meaning of symbol and notation used in the paper.

Symbol/Notaion	Meaning
*j*	$j=\sqrt{-1}$
${\left(\cdot \right)}^{T}$	transpose operator
${\left(\cdot \right)}^{*}$	conjugate operator
${\left(\cdot \right)}^{H}$	conjugate transpose operator
${\left(\cdot \right)}^{-1}$	inverse operator
E[·]	expectation operator
⌈·⌉	upward rounding operator
Tr[·]	the trace of a matrix
⊗	Kronecker product
$\Phi [h_{1},\cdots ,h_{2}]$	get the $h_{1}$ th element to the $h_{2}$ th element from matrix Φ

**Table 3:** Summarized steps of the proposed algorithm.

Step	Operation
Step 1	Construct the CA/OCA and the DOA estimation system model.
Step 2	DOA estimation using conventional algorithm–Capon
Step 3	DOA estimation using the proposed TR-Capon-DOA algorithm
Step 4	DOA estimation using the proposed TR-NS-Capon-DOA algorithm
Step 5	Analyze the performance (including the resolution, accuracy, RMSE, CRLB and computational complexity) of the proposed TR-Capon-DOA and TR-NS-Capon-DOA algorithms with the comparison of conventional Capon mehtod under the condition of different arrangements of array, unknown ES and multipath.
